# Review of interdisciplinary devices for detecting the quality of ship ballast water

**DOI:** 10.1186/2193-1801-3-468

**Published:** 2014-08-26

**Authors:** Goran Bakalar

**Affiliations:** Universiy of Zadar, Croatia, International Maritime technology Consultancy, Split, Croatia

**Keywords:** Neutralization, Ballast, Water, Devices, Detection, Verification, PROMETHEE II analyses

## Abstract

The results of the ship ballast water treatment systems neutralization need to be verified in a transparent and trustful way before the ship enters a port. Some researches and results, explained in this article, confirm a need for a good verification. If there is no good methodology agreed, then it would not be accepted the solution that the BWMC (*Ballast Water Management Convention*) 2004 did protect the sea environment in full meaning. The main problem of ballast neutralization are remaining microorganisms (algae blooms, bacteria) ≥10 and <50. Autonomy of the future ballast water detection device has been explained and newest detection methods analyzed. The ranking analysis has been done thru PROMETHEE II (*Preference Ranking Organization Method for Enrichment Evaluations)* and results were shown by D-Sight software projections.

## Introduction

There are high functioning and low functioning BWTS (*Ballast Water Treatment Systems*). Under the Intermational Maritime Organization (IMO) Ballast Water Convention.

Ballast water will have to be treated once that convention comes into effect (Gregg et al. 2009). Additionally, U.S. regulation independently requires treatment limits to be met and vessel self monitoring (Albert et al. 2013). Numerous BWTS have been type approved by flag administrations to meet the requirements of the IMO ballast water convention (Lloyd’s 2011, Environmental Protection Agency EPA 2011). Newest detection methodologies and belonging tools that could detect treated ship ballast water quality and the contents of it are explained in further text. Since all those technologies are developed more and more every day, the possibility of their use in treated ships’ ballast water quality detection is already existing. When scientific experts or trained port state control officers board a ship and sample, they can be expected to have a higher level of training and expertise. (WDFW Washington Department of Fish and Wildlife 2009). When shipboard crews must sample, sampling methodologies must be simpler or automated. This has been implemented for some discharges other than ballast water, such as bilgewater discharges, which are monitored by automatic oil content monitors (McLaughlin et al. 2014). Automated equipment should do analyses to meet regulatory limits and to be autonomous from ship’s crew to avoid potential crew’s mistakes or not reported BWTS performance failures. Another significant difference of detection tools is coming from the aspect of time needed for ships’ ballast water sample analyses. Ship owners need the shortest time period analyses without possible legal BW quality problems and that implicates automated equipment use. Another importance is size of the tools that could fit ships with lack of the room. The devices have to be resistant to atmospheric influences and to dynamic ships’ movements.

## Material and methods

Material in this research is treated ballast water with the questionable content of algae and bacteria. There are technologies and methods available for detection of those microorganisms. Methods are Pulsed Amplifier Modulated Fluorometry (PAM), Flow Cytometry, Fluorescein Diacetate (FDA), Adenosine Triphosphate (ATP) and automated colorimetry with belonging detection tools.

### Method pulsed amplifier modulated fluorometry

PAM fluorometry (*Pulse Amplifier Modulated Fluorometry*) methodology measures fluorometric character of a particle where Chlorophyll fluorescence originates where the fuel for all life processes is generated (Schreiber 2004). Principle of counting pulse amplified modulation is based on selective increasing fluorescent signals measured by intensive, but very short pulses of measuring light. This technology has been analyzed by many scientists in last decades. Good results in water analyses were achieved. Technologically, the footprint and computing has progressed so much that PAM fluorometry can be taken into consideration for the final selection of potential detection method of unwanted microorganisms detection.

Views, achievements and possibilities of PAM using for the analysis of phytoplankton as the foundation of today’s achievements, were presented 1984. (Jameson et al. 1984). During research of fishing crime impact it was proved that is possible to use PAM methodology for cyanide findings (Jones et al. 1999).

“Cyanide fishing”' in that case has been used to supply a rapidly expanding restaurant-based market for live reef fish, primarily rock cod and grouper (Epinephelus spp.), coral trout (primarily Plectropomus spp.), barramundi cod.

(Cromileptes altivelis), Napoleon wrasse (Cheilinus undulatus) and also lobster (Panulirus spp) (Johannes and Riepen 1995).

Characteristics of PAM device with the requirements for additional training and accurate maintenance and handling are aggravating circumstances in deciding on the possible use of this technology by some of the crew members in the event that one wants to analyze ballast on board before the ship approaches port. However, it is not impossible the use of such devices by representatives of port authorities if we want to achieve a clearer picture of the quality of ship ballast on a ship.

### Method flow cytometry

Flow cytometry allows easy recognition of the different groups in the sample and quantification of their abundance as well as their optical properties (size, pigment) - even the detection of a few rare cells from within a high number of cells from a blooming species (Dubelaar and Gerritzen 2000). Simply, this is an automated way of detection and microscope is a tool for same purpose, but is not automated. Scanning of 10,000 scans per second is one of the advantages of this tool (CytoBuoy 2013).

The flow cytometer could be fixed on ships, under water and on moorings, and should allow a high level of autonomous operation combined with high speed and high throughput (CytoBuoy 2013). Results of flow cytometry measuring have shown the possibility of using flow cytometry for detection of microorganisms size less than 10 μm, which is very important in the event that the obligations of the treatment of this size microorganisms may be point of future amendment to the Convention on the Management of Ships’ Ballast Water and Sediments from 2004 (Van Der Star et al. 2011). Size of organisms smaller than 10 μm, precise size of 9 μm, showed the ability of re-growth or resurgence after treatment. It happens when the treated water get returned back into the daylight or in natural seawater (Liebich et al. 2012.

A flow cytometer could operate quite autonomously, almost without crew impact to the results of detection and data could be filed securely. The instrument could be automatically stained and flushed by fresh water prior to use. It is just needed to calibrate fixed flow cytometer after each use and that could be done easily by a crewmember (Chief Electrician) and ship’s computer (Bakalar 2013).

### Method FDA/ATP

This detection method has been analyzed by scientists since a few decades ago. Fluoroscein Diacetate Hydrolisis, as a complete microorganisms activity, is a good measurement of total biomass if the microorganisms incubation has been done in the shortest possible time period before the measures were taken (Schnurer and Rosswall 1982). It was a successful use of fluorescein diacetate in purpose to find out quantity of bacteria and funguses in a sample of protist particle (Schnurer and Rosswall 1982).

Protists are amebas and algae. That is a proof for possibility of using this methodology for bio -invasive microorganisms detection in treated ballast water.

This technology, analysis and detection of microorganisms in ballast water is a measure of the total energy of life in a sample of treated ballast water or direct microscopic observation to enumerate viable organisms ≥10 and <50 μm in minimum dimension (Steinberg et al. 2011).

ATP (*Adenosin Triphosphate*) is molecule produced by all living organisms and is reflected by measuring the amount of universal or total living biomass energy measured in a sample, in this case, the ship’s ballast water. The analysis are done in a period of 3 minutes or more, and the equipment is easy to operate. Another system analysis and detection is the measurement of the total enzyme activity in a sample of ballast water. Enzymes FDA (*Fluorin Diacetate*) are also as ATP, produced and secreted from living organisms and measurable indicators of living biomass in a sample of ballast water.

In both systems of measurement, after the ship’s ballast water treatment, device identifies the remains of living organisms through indicators that represent the two technologies. Thus it can be said that a system of ship ballast water treatment has, correctly or incorrectly, sufficiently or insufficiently, neutralized unwanted organisms in ship ballast water. The water analysis, to check live biomass on board the ship, has to be performed by the recirculation of the ballast tank by tank in purpose to achieve effective and credible measurement. The use of such a device by one of the crew members would require an additional training.

This methodology has been proposed by research team in NIOZ institute in the Netherlands. It could be used to check the quality of the treated ballast water on ships by the port authority. According to the research results (Van Slooten 2012) of these devices use for the purpose of the primary analysis of living biomass in the sampled fluid, it was advised that the port authority officer may order additional control of ship ballast water if the results of the FDA or ATP analysis were unacceptable. Additional controls would then be operated in mainland microbiological laboratory. Analysis of samples would be done with lasting several hours waiting for the results. It might easily happen that a ship has to leave the port and stay at the anchorage for further analyses and legal work.

ATP measurement system that was the subject of the research (Van Slooten 2012) at the institute NIOZ is not automatic and it is not autonomous. It requires a higher level of training and an operator to operate the device and measurement. The operator would be, if it is not a representative of the port authority, a member of the crew. Questionable is also the size of the legal consequences caused measuring enzyme and measuring energy live biomass in the sample ballast water because there is no separation of the measured size of the type and size of organisms, so it is possible that the energy and activity levels relate to the part of the organisms smaller than 10 microns and that is not covered by regulation D-2 of the Convention. Also, some measured energy remains even with the dead microorganisms and it is not possible to separate calculated amount from viable to dead.

### Method automated colorimetry

Automated colorimetry is an automatic microbiological system for identification of microorganisms using colorimetric reagents and additional necessary equipment. Colorimetric optical device has been in use during analysis done in project number 3137, Istanbul University, the Faculty of Fisheries, Department of Marine Biology, Canakkale Onsekiz Mart University, Canakkale, Turkey. Two years were analyzed samples taken from the ballast tanks of ships passing through the Marmara Sea. It was proven a bacterial resistance to common antibiotics, and their survival for a longer time in the ship’s ballast tanks.

During the trial, in year 2010, 50,871 ships sailed through Istanbul’s passage into the Black Sea. This project dealed with the frequency of beta-lactam antibiotic resistant bacteria, in order to draw attention to the risk to human health that comes by various ballast operations of ships passing through the area.

Samples were isolated from the ballast tanks into sterile glass bottles and then the samples were tested using automatic micro identification of micro devices VITEK 2 Compact 30, Biomereux (VITEK 2013). As per understanding of the autonomy, measurability features and automatic use of a detection system for quality and quantity detection, colorimetric analysis of the ballast sample optical properties are considered automatically insufficient because it requires the crew assistance at the saving colorimetric card time period. Regardless of any other analysis required, this methodology cannot achieve acceptable level autonomy system in relation to the ship’s crew. In fact, it is needed to avoid the possibility of obstruction this kind of devices by crew members from the experience point of view from the ships where the Oil Discharge Monitor was used to monitor discharge of oil. That system was often by-passed so it would not exist any possibility of the crew impact during the device detection operation.

### Multi-criteria analyses of the methods

#### Criteria

Purpose of this analyses is to get an autonomous detecting tool with good performances and the ship has to be ballast checked out before entering the port. In that way there is no time loss for analyses and no legal work stoppages (Bakalar 2013). From the point of eligibility for monitoring the quality of purified BBV all systems were analyzed according to the following criteria, shown in Table 1:

**Table 1:** **Parameters for multi-criteria analyses**

SYSTEM	Automation	Autonomous	Organisms size	Speed time	Price USD
Flow cytometry	Yes	Yes	<10 micron	10,000 per second	$ 18,000 to 80,000
FDA/ATP	partial	No	Energy of all organisms in a sample	3 or more minutes to detect enzimes quantity	FDA $ 450
Automated colorimetry (VITEK)^1^	partial	No	<10 micron, but not all organisms	15 minutes for each test	$ 32,000 to $ 151,000
PAM floumetry	partial	No	10 <50 micron	10 seconds to identify and detect chlorophyle	$ 58,000 to $ 93,000

1. Automatic operation
2. Autonomy of operation
3. Detection of organisms by size (quality control of the results of BWTS performance)
4. Detection speed
5. Price of devices

### Analysis

The criteria is from different areas: economy, performance, scanning speed, autonomy related to human. That implicates the possibility of use **PROMETHEE II** multi-criteria analyses.

PROMETHEE method was designed to analyze parameters of criteria including alternative choice of specific criteria. In a mathematical way, that is:


where ***A*** means final decision of ***n*** alternatives of multi-analysis criteria from ***f***_1_ *to* ***f***_*k*_, chosen for ranking ***f***_***j***_(**a**) is evaluation of choice a thru criteria ***f***_***j***_**:**

where ***w***_***j***_ > **0** is the criteria weight ***f***_***j***_ (more important criteria is given more weight). ***V(a)*** is sum of all ***a*** alternatives from ***a***_***1***_ to ***a***_***n***_**.**

In PROMETHEE I partial ranking, ***φ***^+^ shows how much one criteria prefers the other criteria:


Other option ***φ***^-^ shows weakness of one criteria against the other criteria:


“***a***”prefers “***b***” only if:


and also:


In PROMETHEE II complete ranking, the ranking criteria flows in opposite directions because of the different views which are not touching each other; it means:

“***a***” prefers “***b***” only if:


Because of that reason, in this criteria analyses was used complete ranking PROMETHEE II.

**Figure 1:**
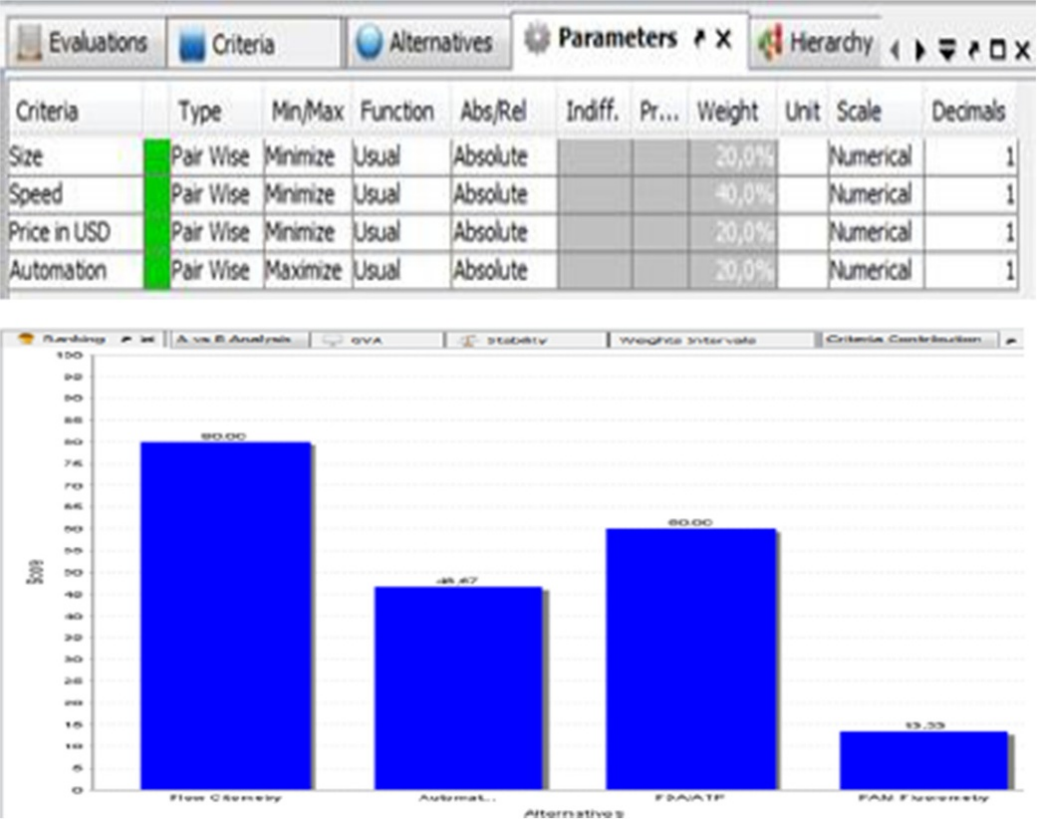
**Multi-criteria analyses PROMETHEE II, visual D-Sight results.**

D-Sight GAIA computerized visual method, as seen in the pictures, is also shown in the results of ranking as the most appropriate system. That is a visual projection of PROMETHEE analysis. Three parameters were used for detection criteria alternatives, currently the most researched. The most important parameter that determines the acceptability of the existing autonomy belongs exclusively to the flow cytometry, and that is why was not set as a criteria for multi-criteria analysis to achieve gender criteria in the analysis. Size of organisms is also a very important factor which is lately increasingly researched because of the frequent attitude of scientists that were complaining of not included organisms smaller than 10 microns in the Convention of the 2004. (Van Der Star et al., 2011; Liebich et al. 2012). Criteria “Price unit” is the biggest advantage of FDA/ATP detection, but the device of that methodology is not autonomous enough and device handling and detection would be left to one of the crew members, which is unacceptable in this case, where an autonomous quality control of ballast water is research aim. The criteria of “speed detection” is the advantage of flow cytometry, but the most important quality of the flow cytometer is just autonomy of the detection. All those parameters were weighted as per above comments. Figure 1 shows the results of the ranking in accordance with previous explanations.

## Results

**Figure 2:**
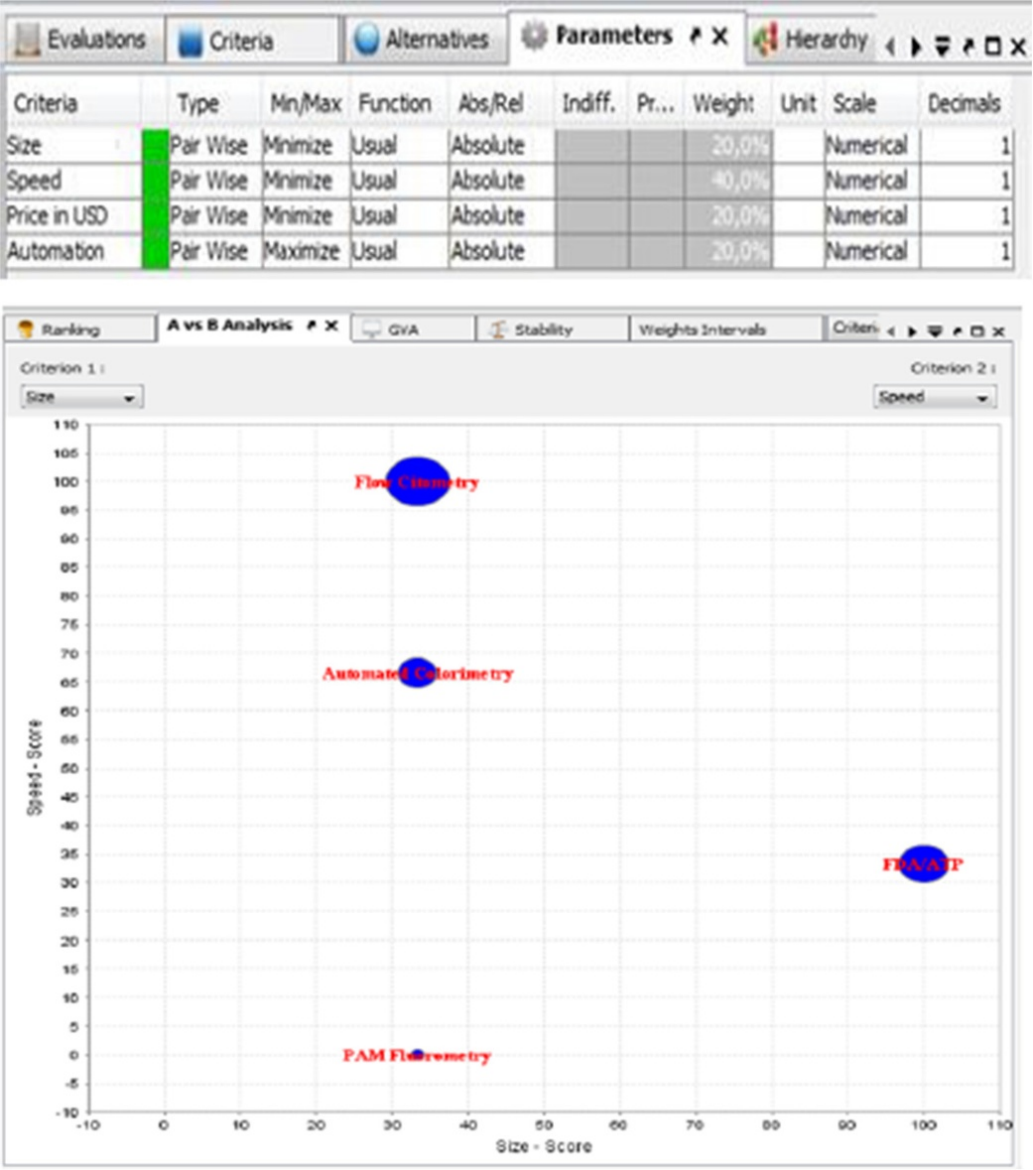
**Visual “A vs. B” analysis result projection.**

Even without weighting of “autonomy” criteria (because it was with flow cytometry only), flow cytometry scored best results. The clarification presented in Figure 2, and the nodes are the result of analysis. It gives the highest preference to flow cytometry as well. FDA/ATP detection methodology is, nevertheless is not autonomous, quality indicator of whether the ballast water treatment was well done on the ship and the quantity of detected enzymes or bio-energy could obtain data that are useable and could be justified by the decision to initiate the port authorities to claim additional analysis. Spare parts from different manufacturer other than the one that has produced the equipment might not have work so well in every unit. ATP systems, including reagents, are designed to work like a unit, especially since it was related to the reproducibility of the results in the presence of plant chemicals such as those for sanitary. Therefore, considering the value of ATP test equipment, consistency and availability of materials are essential for smooth operation and use of equipment. Finally, ATP testing equipment requires a quality operator. The operator uses the equipment and support to the manufacturer or retailer. Without proper training, education and technical support, investment in ATP system can be unproductive, especially when used on the ship.

However, analysis of the research and the most comprehensive detection and speed required for such coverage are on the best ranked criteria, which is flow cytometry with the technology reaching most microorganisms detected in the shortest period. That “A” vs. “B” analysis result projection is on Figure 2.

**Figure 3:**
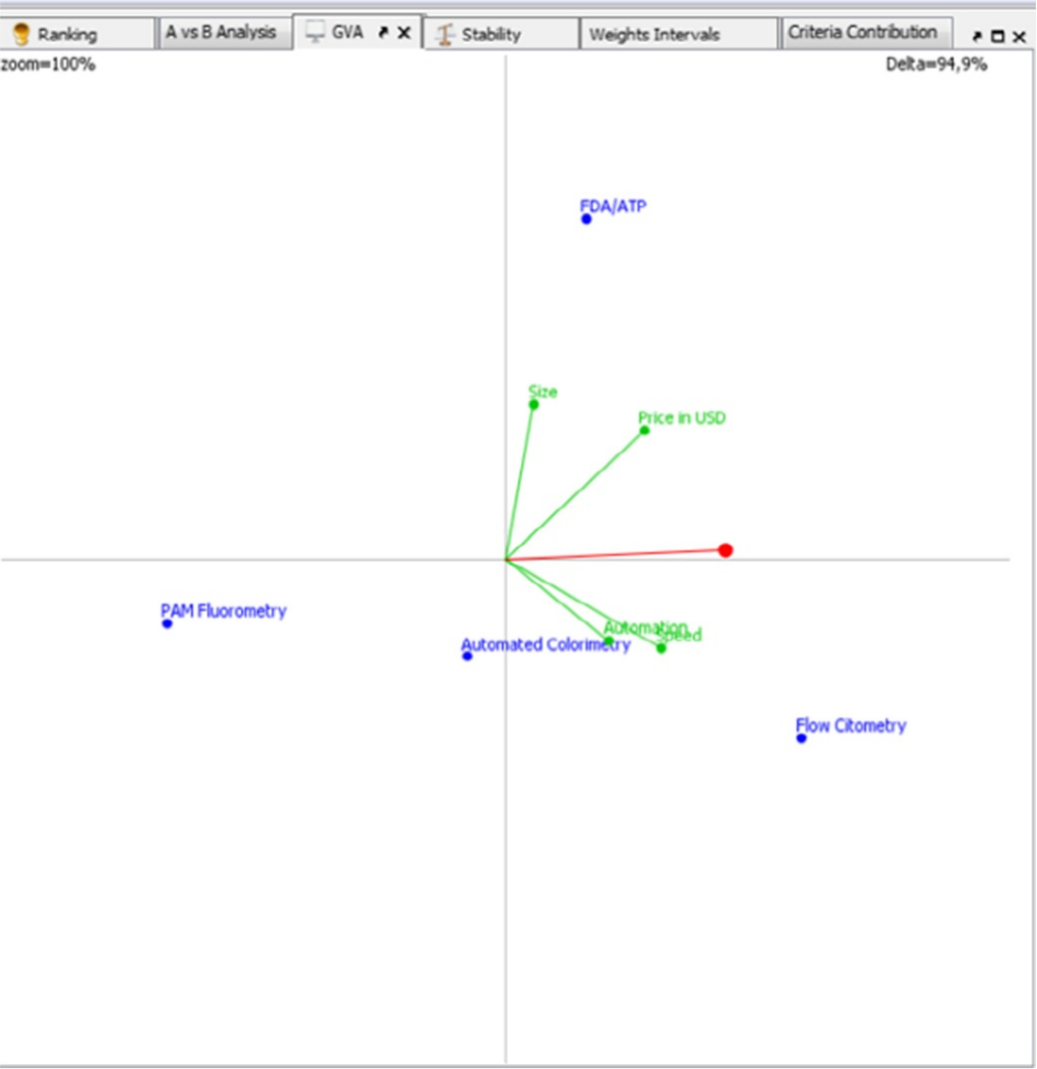
**D-Sight GVA projection of results.**

Results of the multi-criteria analysis, as it is also evident on GVA (Global Visualisation Analyses) Figure 3, the flow cytometry technology and flow cytometer were selected for monitoring and control of the results of the operating system for the treatment. FDA/ATP detection technology has achieved good success in this computational analysis because of the price of 450 USD for a single device detection. Anyway, the closest to the “ideal node” was flow cytometry determining the quality of the treated ballast water through automatic detection of unwanted microorganisms.

**Figure 4:**
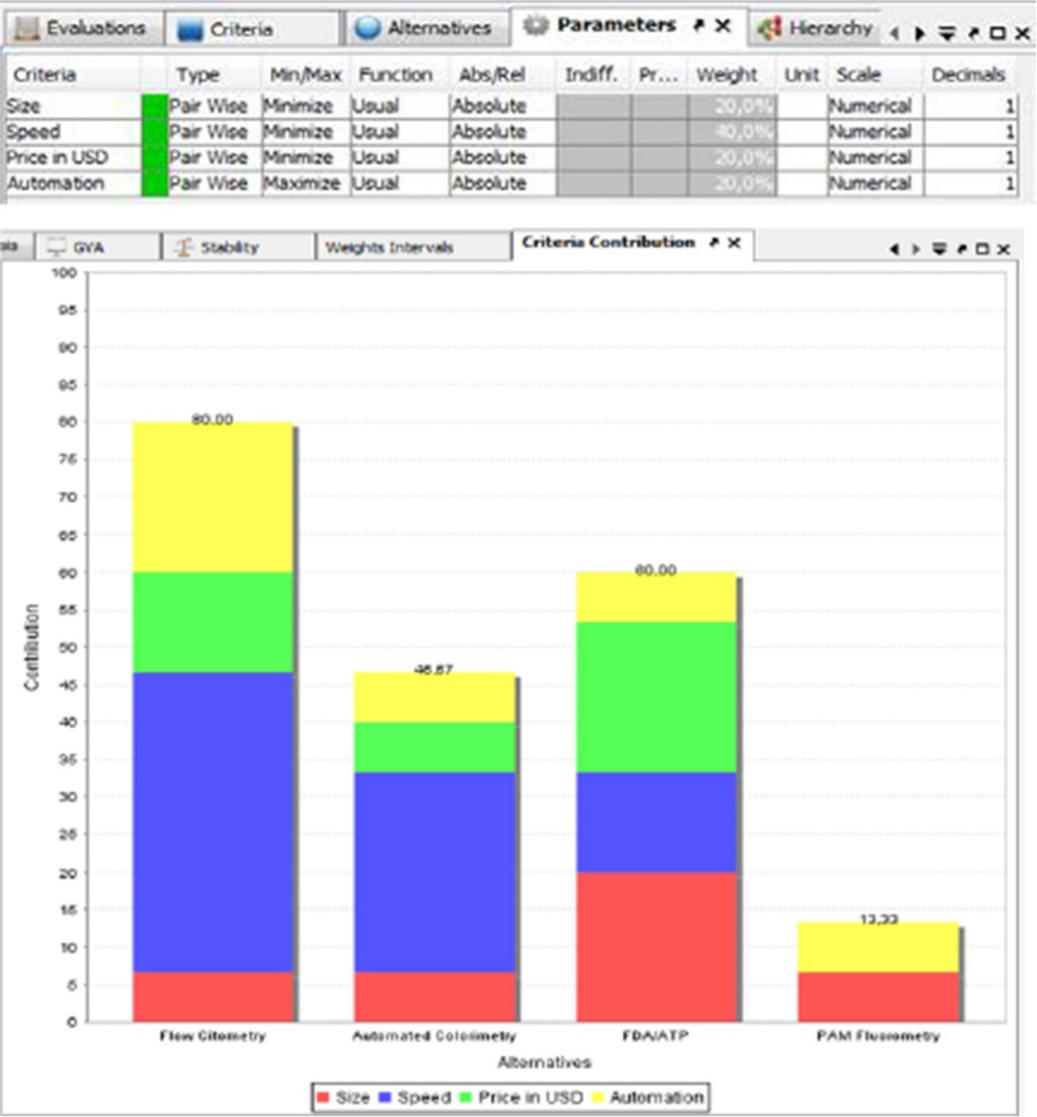
**D-Sight of all parameters.**

This presentation of the analysis results indicates that there is a lot of conflict, since, in this scenario, all of the criteria is on the right side. Two most suitable detection systems are on the further distance, and these are flow cytometry and FDA/ATP. All parameters are colored on Figure 4.

## Discussion

In the treated ballast water, after the treatment of most of neutralization systems, remains, beside to organisms ≥10 and <50, a significant amount of organisms smaller than 10 microns. The results of research and detection by flow cytometry indicated that 80% of all existing phytoplankton in seawater are microorganisms smaller than 10 microns (Veldhuis and Kraay 2000). Required detection quality (indicative or compliance monitoring detection) from aspect of microorganisms size, speed of analyzes, period of analyses and needed crew education is analyzed in this review and it is preferred on flow cytometery and FDA/ATP devices. Also, one of the most important factors and enforceability aim of this analysis is autonomous operation in relation to the ship’s crew. This is the preferred advantage of flow cytometry as, in possible future analyzes, the entire process of detection could be done remotely without being affected by any of the crewmember (Bakalar et al. 2011).

## Conclusion

According to the multi-criteria results, as a scientific method, there is not existing any single technology that could be capable of direct detecting, counting and analyzing BWTS quality except flow cytometry. This technology also allows fast sample processing and it is not limited with the speed of the particle movement. The most important, the operation of this technology tool, flow cytometer, could be operated automatically and autonomously from the ships’ crew impact, in case that a BWTS operates improperly and the crew didn’t report the malfunction. Also, microorganisms smaller than 10 microns could become an important fact (Cangelosi 2011) in the future and automated autonomous compliance monitoring with flow cytometers would play a significant role in their detection.
